# Space Radiation-Induced Alterations in the Hippocampal Ubiquitin-Proteome System

**DOI:** 10.3390/ijms22147713

**Published:** 2021-07-19

**Authors:** Alyssa Tidmore, Sucharita M. Dutta, Arriyam S. Fesshaye, William K. Russell, Vania D. Duncan, Richard A. Britten

**Affiliations:** 1 Department of Radiation Oncology, Eastern Virginia Medical School, 700 W. Olney Rd., Lewis Hall, Norfolk, VA 23507, USA; alyssa.l.tidmore@gmail.com (A.T.); FesshayAS@evms.edu (A.S.F.); v.d.miller.vm@gmail.com (V.D.D.); 2 Department of Microbiology and Molecular Cell Biology; Eastern Virginia Medical School, Norfolk, VA 23507, USA; sucha227@gmail.com; 3 Leroy T. Canoles Jr. Cancer Research Center, Eastern Virginia Medical School, Norfolk, VA 23507, USA; 4 Center for Integrative Neuroinflammatory and Inflammatory Diseases, Eastern Virginia Medical School, Norfolk, VA 23507, USA; 5 Department of Biochemistry and Molecular Biology, University of Texas Medical Branch, Galveston, TX 77555, USA; wirussel@UTMB.EDU

**Keywords:** space radiation, hippocampus, proteome, ubiquitin, spatial memory, cognition

## Abstract

Exposure of rodents to <20 cGy Space Radiation (SR) impairs performance in several hippocampus-dependent cognitive tasks, including spatial memory. However, there is considerable inter-individual susceptibility to develop SR-induced spatial memory impairment. In this study, a robust label-free mass spectrometry (MS)-based unbiased proteomic profiling approach was used to characterize the composition of the hippocampal proteome in adult male Wistar rats exposed to 15 cGy of 1 GeV/n ^48^Ti and their sham counterparts. Unique protein signatures were identified in the hippocampal proteome of: (1) sham rats, (2) Ti-exposed rats, (3) Ti-exposed rats that had sham-like spatial memory performance, and (4) Ti-exposed rats that impaired spatial memory performance. Approximately 14% (159) of the proteins detected in hippocampal proteome of sham rats were not detected in the Ti-exposed rats. We explored the possibility that the loss of the Sham-only proteins may arise as a result of SR-induced changes in protein homeostasis. SR-exposure was associated with a switch towards increased pro-ubiquitination proteins from that seen in Sham. These data suggest that the role of the ubiquitin-proteome system as a determinant of SR-induced neurocognitive deficits needs to be more thoroughly investigated.

## 1. Introduction

NASA’s planned mission to Mars will take about three years to complete, during which time astronauts will be exposed to ~13 cGy Space Radiation (SR) each year [1]. SR is composed of highly energetic charged ions: protons, helium nuclei, and high-mass and energy (HZE) (Z ≤ 28, nuclei [2]). It has been calculated that roughly a quarter of the estimated 43 million hippocampal neurons within the brain will be hit directly hit by one or more particles with Z ≥ 15 on a deep space mission [3].

The hippocampus is essential for multiple neurocognitive process, including associative recognition memory [4] and the formation and retrieval of both new and old episodic memories [5,6]. Ground-based rodent studies have shown that exposure to <20 cGy SR impairs performance in several hippocampus-dependent cognitive tasks, including spatial memory in both rats [7,8,9,10,11,12] and mice [13,14,15], while, at the cohort level, SR exposure impairs spatial memory performance; at the individual levels, some irradiated rats maintain a spatial memory performance comparable to that seen in the sham-irradiated rats, while others have severely compromised performance [10,11,12]. These data suggest that some individuals are able to ameliorate the deleterious effects of SR while others are unable to do so. This bifurcating response of neurocognitive processes to SR exposure has important consequences for risk assessments but also provides a unique opportunity to establish the impact of SR on neurophysiology, and the subsequent adaptive responses associated with the impairment or preservation of neurocognitive performance. SR exposure induces multiple changes in the functionality of hippocampal neurons: changes in the complexity of the dendritic tree in the hippocampus [15,16,17,18], long-term suppression of glutamatergic transmission and NR2A receptor levels in the hippocampus [19], homeostatic synaptic plasticity [18,19,20,21,22,23], as well as autophagy and persistent oxidative stress [24]. Recent studies also suggest that SR-induced neurocognitive decrements may be regulated by non-neuronal components, with the activation status of the microglia in the first few days after exposure being important [25,26,27]. Collectively, these studies indicate that SR exposure alters numerous processes within the hippocampus, and that no single process will likely dominate the cognitive performance of an irradiated individual. Taking all these factors into consideration, it seems likely that a systems biology approach will be necessary to identify why some individuals retain a functional spatial memory, while others have impaired performance after SR exposure.

We have previously employed a robust label-free mass spectrometry (MS)-based unbiased proteomic profiling approach to characterize the composition of the hippocampal proteome in juvenile [28] and adult [29] male Wistar rats exposed to ≤20 cGy of 1 GeV/n ^56^Fe. These studies identified proteins whose expression are altered with respect to (1) radiation exposure and (2) impaired spatial memory performance. Nearly a quarter of the proteins found in the hippocampus of adult sham rats were lost or had reduced expression in the irradiated hippocampus [29]. These data are consistent with the SR-induced changes in the DNA methylation status within the hippocampus [30,31,32,33,34]. The biological significance of these methylation changes is quite pronounced since SR-induced loss of hippocampal-dependent memory updating and LTP within the hippocampus were reversed by the use of histone deacetylase 3 inhibitors [35]. 

While SR-induced changes in DNA methylation may be an important factor in influencing the composition of the hippocampal proteome, protein abundance is not only determined by transcriptional activity, but also by translation rates, sequestration of proteins into complexes, as well as protein turnover and degradation. SR exposure upregulates several components of the proteasome/protein degradation pathway in the hippocampus [28], and also alters protein homeostasis, with marked changes in ubiquitination [36]. While such SR-induced changes in ubiquitin-mediated cellular protein homeostasis will undoubtedly have profound effects on neuronal physiology and functionality, the ubiquitin-proteome system (UPS) plays a key role in regulating memory formation and consolidation [37,38,39]. Thus, differences in the UPS may serve to modulate the impact of SR-induced changes in neurotransmission on neurocognitive performance, and may explain the poor correlation between these parameters in our previous studies [18,21]. We thus established the composition of the hippocampal proteome in adult male rats exposed to 15 cGy 1 GeV/n ^48^Ti ions and identified protein signatures that are biomarkers of (1) Ti exposure and (2) spatial memory performance, and whether these biomarker proteins are involved with the UPS. 

## 2. Results

The spatial memory performance of the retired breeder (about nine months at the time of irradiation) rats was established at 90 ± 14 days post irradiation using the Barnes Maze. Each rat was tested on the Barnes’ Maze over a three-day period, with two tests per day. A key element of the Barnes Maze is that rats progressively learn and recall the location of the escape box, as measured by the time to find its location i.e., escape latency (ESL). 

For each rat, an individualized metric of their relative increase (or decrease) in performance between days 1 and 3 of testing i.e., the Relative Escape Latency (REL3) was calculated (where the Day 3 ESL for a specific rat is expressed as a decimal fraction of its Day 1 ESL). The REL3 for rats that improve in spatial memory between days 1 and 3 is less than unity. Alternatively, if spatial memory is impaired by Day 3, the REL3 remains near unity or becomes greater than unity. 

**Figure 1:**
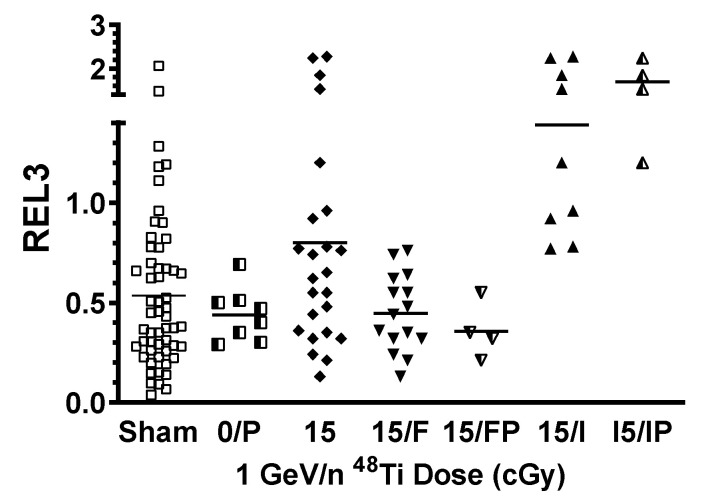
Effect of 1 GeV/n ^48^Ti-exposure on the spatial memory performance of individual rats. Individual REL3 values for sham-irradiated rats (squares) or rats exposed to 15 cGy 1 GeV/n 48Ti (diamonds and triangles); horizontal bar denotes Median value of the REL3 values within a cohort. Cohort abbreviations: Sham: Sham-irradiated rats; 0/P: Sham-irradiated rats used for proteomic analysis, 15: all rats exposed to 15 cGy 1 GeV/n ^48^Ti; 15/F: all rats classified as having a functional spatial memory; 15/FP: 15/F rats used for proteomic analysis; 15/I: all rats classified as having impaired spatial memory; 15/IP: 15/I rats used for proteomic analysis.

The mean REL3 for the entire population exposed to 15 cGy ^48^Ti (0.81 +/− 0.12, *n* = 24) was significantly higher (*p* = 0.046, Mann–Whitney) than the mean REL3 of the sham-irradiated rats (0.53 +/− 0.05, *n* = 55). Rats were classified as having impaired spatial memory (15/Impaired) if their REL3 value was greater than 0.7. The distribution of the REL3 values within each cohort is shown in Figure 1. Representative rats from each cohort (Sham-irradiated: 8 (REL3 < 0.69), 15/Functional: 4 (REL3 < 0.55), 15/Impaired: 4 (REL3 > 1.1 < 2.5)) were selected for subsequent proteomic analysis, the REL3 values for those rats are separately depicted in Figure 1. 

**Figure 2:**
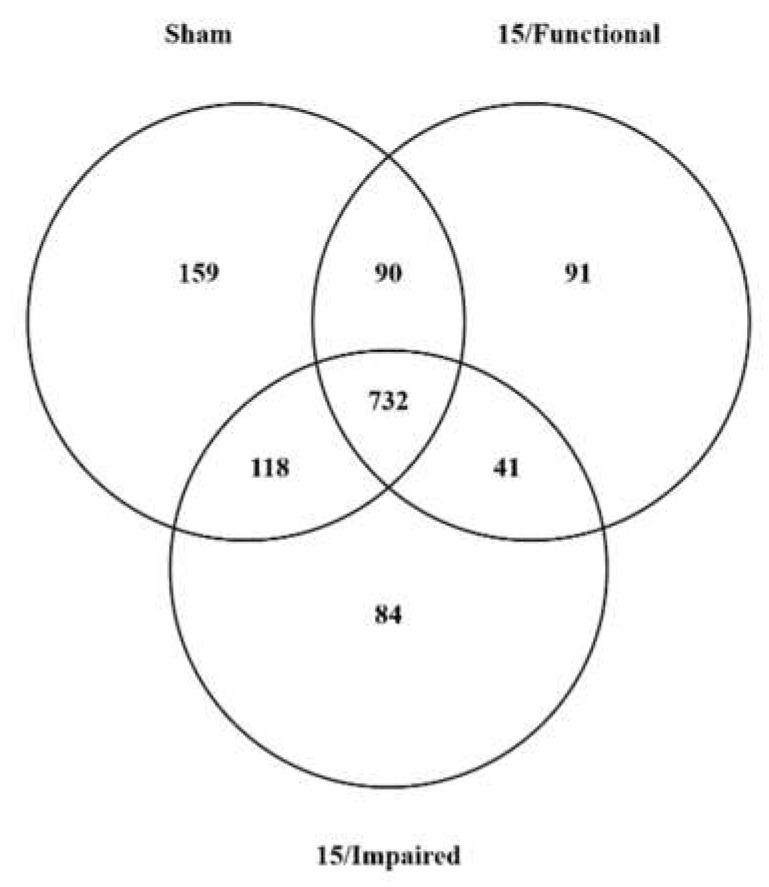
Venn diagram of the number and distribution of proteins identified within the hippocampus of the Sham, 15/Functional, and 15/Impaired rat cohorts. Numbers represent the number of proteins detected in each cohort.

An analysis of the composition of the hippocampal proteome of the representative rats from each cohort (Sham-irradiated (*n* = 8), 15/Impaired ((REL3 > 1.1 < 2.5) (*n* = 4)), 15/Functional ((REL3 < 0.55) (*n* = 4)), revealed that there were 974–1099 proteins that satisfied our vigorous inclusion criteria (quantifiable in >66% of technical replicates, and present in >66% of the biological replicates) in the various cohorts of rats (Sham-functional: 1099; 15/Functional: 954; 15/Impaired: 974). A complete list of the identified proteins within each cohort is provided in the Supplemental Table S1 There were 732 proteins that were detected in all three cohorts, hereafter referred to as “common” proteins (Figure 2). 

There were 159 (88 fully mapped) proteins that were detected in the proteome of the sham rats but were not detected in the irradiated rats (irrespective of their spatial memory performance status), hereafter referred to as “Sham only” signature proteins. The Uniprot accession numbers of the 88 fully mapped “Sham Only” protein are listed in Table 1. The gene/protein names of all proteins detected in the study are listed with the appropriate Uniprot numbers in the Supplemental Table S1.

While these proteins are classified as “sham only” proteins, they are also technically negative biomarkers of Ti exposure. There were also 41 positive biomarkers of Ti exposure, i.e., were detected in both the 15/Functional and 15/Impaired cohorts, but not in the sham proteomic profile, hereafter referred to as “Ti exposure marker (TEM)” signature proteins. The 20 fully annotated and reviewed TEM proteins are listed in Table 2.

In addition to the TEM proteins, the proteome of the Ti-exposed rats contained other unique proteins that reflected the spatial memory performance of the irradiated rats. There were 84 proteins that were found only in rats who had impaired spatial memory performance (15/Impaired), hereafter referred to as “spatial memory impairment (SMI)” signature proteins. The 37 fully annotated and reviewed SMI proteins are listed in Table 3. There were an additional 24 upregulated TEM proteins (italicized in Table 3).

The hippocampal proteome of the irradiated rats that exhibited sham-like spatial memory performance (15/functional) rats was characterized by the presence of 91 uniquely expressed proteins, hereafter referred to as “preservation of spatial memory impairment (PSM)” signature proteins. The 41 fully annotated and reviewed PSM proteins are listed in Table 4. There were an additional 14 upregulated PSM proteins (italicized in Table 4).

Our unique ability to identify proteomic signatures that are associated with a phenotypic endpoint (spatial memory performance) provides an opportunity to investigate how SR exposure can lead to spatial memory impairment in some rats (15/Impaired) and not others (15/Functional). As observed in our previous studies on Fe-induced changes in the hippocampal proteome [28,29], there were significant Ti-induced changes in the expression of proteins involved with neuronal homeostasis, axonogenesis, pre-synaptic membrane organization, G-protein coupled receptors oxidative damage response, calcium transport, and signaling. One notable change was the unique presence of Glia-maturation factor B within the PSM signature. 

Gene Ontology (GO) enrichment analysis (using FunRich) of the six protein signatures (i.e., “Sham only”, “TEM”, “SMI”, “PSM”, “Impaired” Phenotype (TEM and SMI), and “Functional” (TEM and PSM) proteins) identified five processes that satisfied our inclusion criteria: GO:0044877-protein-containing complex binding; GO:0042802-identical protein binding; GO:0046872-metal ion binding; GO:0005524-ATP binding; and GO:0019901-protein kinase binding. Over 30% of the SMI proteins were identified as having an important role in the GO:0042802-identical protein binding and GO:0005524-ATP binding processes (Table 5). 

The overlap with the ubiquitin-proteome system (UPS) UniProt database for each of the six protein signatures, revealed that roughly a quarter of the proteins in the TEM, SMI, and Impaired signatures have a direct role in the UPS (Table 6). The Sham signature was unique in that it was the only one to contain proteins that exhibited deubiquitinase (DUB) activity, with two deubiquitinases (Q4VSI4/Usp7; Q9R085/Usp15) being absent in all irradiated cohorts. The UPS is a key regulator of protein homeostasis within the cell; we utilized a simple ubiquitin homeostasis algorithm to determine if there were any indications of SR-induced changes in UPS functionality within the various signatures. The Sham signature yielded a pro-de-ubiquitination weighting (−4), whereas both the Functional (TEM and PSM) and Impaired (TEM and SMI) signatures yield a pro- ubiquitination weighting (7.5 and 8.5 respectively). The TEM signature in isolation had a +2 weighting factor.

## 3. Discussion

The present study is one of only a few [28,29,40,41,42,43] to utilize a bottom-up proteomic profiling strategy to identify changes in the hippocampal proteome that occur after radiation exposure. The proteomic profiles generated in this study are essentially a snapshot of the functional status quo within the hippocampus at the time of sampling (approximately three months after exposure). There are unique protein changes that are observed in all irradiated rats (loss of sham only and de novo expression of TEM proteins). These changes may have occurred as an immediate response to SR exposure, or may be the result of compensatory responses invoked over time in response to the SR exposure. Superimposed on these “exposure” related changes, there are further protein signatures that are found in rats that have functional (PSM proteins) or impaired spatial memory (SMI proteins) performance. A unique feature of our study has been the identification of protein signatures (biomarkers) that are associated with a specific phenotypic (spatial memory functionality) endpoint, in this case spatial memory performance, assessed just prior to recovery of the brain tissue. Thus, there is a reasonable likelihood of a casual association with the observed proteomic (PSM and SMI) changes to spatial memory performance. However, as previously reported [28,29], there are complex changes in the composition of the proteome that will most likely require advanced analysis (such as identification of reciprocal up- and downregulation of multiple proteins/pathways) to identify which of the observed changes have phenotypic significance. 

One of the most striking features of the proteomic profiling was that ~14% of the proteins found in the hippocampus of sham rats were lost in the irradiated hippocampus (Figure 2, Table 2). While these proteins have been classified as “sham only” signature proteins (since they were only detected in unirradiated rats), these proteins are also technically negative biomarkers of Ti exposure. Reduced expression of hippocampal proteins may be a hallmark (biomarker) of SR exposure since we have previously observed a SR (Fe)—induced loss of protein expression [28,29]. SR (Si) exposure elevates DNA methylation levels in the hippocampus [30], which would certainly be consistent with a reduction in protein expression. However, SR-induced DNA methylation levels would appear to not be the sole determinant of the observed changes in the hippocampal proteome, given that there were many additional (and complex) changes that occur in the hippocampal proteome of SR-exposed rats, including the upregulation (or de novo) expression of many proteins. The functionality of the hippocampus in SR-exposed rats may be reflected by the changes in the expression of the TEM proteins. In our previous studies [28,29], these SR exposure markers included many involved in neuronal homeostasis, axonogenesis, pre-synaptic membrane organization, G-protein coupled receptors oxidative damage response, calcium transport, and signaling. There are further proteomic changes in the hippocampus of irradiated rats that are associated with the loss of spatial memory performance, while the proteome of rats that have been able to preserve spatial memory performance have another proteomic signature. Notable amongst the PSM signature proteins was the unique presence of Glia-maturation factor B (Table 4). Several studies have demonstrated that SR-induced behavioral and memory deficits are associated with altered microglial activation [25,26,27]. We have included the proteomic expression data from our various rat cohorts (as Supplementary Material) for others to mine using the new generation of bioinformatics approaches to determine the full spectrum of changes present in these rats. However, it should be noted that the proteomic signatures of the various cohorts identified in this study do need orthogonal validation. While Western blotting is frequently used for such purposes, the linearity of antibody-generated signals needs extensive validation to get truly quantitative data. A more feasible approach with the more widespread use of “Orbitrap” mass spectrometers would be to use multiple reaction monitoring (MRM)/parallel reaction monitoring (PRM) of specific peptides from the proteins of interest [44]. 

Collectively, the specific upregulation (or de novo) expression of multiple proteins suggest that SR-induced methylation cannot be the sole determinant of SR-induced changes in the hippocampal proteome. Protein expression is a complex balance between transcriptional/translational activity and protein turnover/degradation. The maintenance of protein homeostasis is an important role of the ubiquitin-proteasome system, which is responsible for roughly 80% of all intracellular protein degradation [45]. SR exposure has recently been shown to alter the ubiquitin-mediated protein homeostatic response in cultured neural cells, associated with an elevated number of ubiquitination-site linkages [36]. Nearly a quarter of the proteins in the TEM, SMI, and Impaired signatures identified in this study have a direct role in the UPS (Table 6). Furthermore, only the Sham signature contains proteins that exhibited deubiquitinase activity, with two deubiquitinases (Q4VSI4/Usp7; Q9R085/Usp15) being absent in all irradiated cohorts. While both of these hydrolases impact a wide range of ubiquitin-dependent processes, the Usp7 protein is of special interest in the context of SR-induced loss of neurocognition. The Usp7 protein involved in maintaining DNA methylation, and the loss of Usp7 in the irradiated rats may thus be related to the SR-induced epigenetic changes [30]. Usp7 also deubiquitinates the repressor element 1 silencing transcription factor (REST), thereby stabilizing REST and promoting the maintenance of neural progenitor cells [45]. Radiation-induced changes in the Usp7-mediated deubiquitination of REST could thus account for the neural precursor-cell dysfunction reported following low-LET radiation exposures [46]. REST also plays an important role in the shaping of the synaptic output of adult NMDAR dependent neurons [47], so the loss of neurocognitive function following SR exposure could in part be mediated via Usp7-mediated deubiquitination of REST.

Our data would suggest that the SR-induced changes in UPS in cultured neuronal cells [36] also occurs in vivo, at least within the hippocampus of rats exposed to 15 cGy Ti ions. However, while the UPS system does play a key role in “bulk” protein homeostasis, UPS-mediated activity-dependent protein degradation is required for memory consolidation [48], consolidation of spatial memory [49,50], contextual fear conditioning, and inhibitory avoidance [37,38,51,52]. The loss of deubiquitinase activity after Ti exposure could certainly contribute to the loss of spatial memory performance. The very simplistic ubiquitin homeostasis algorithm that we developed has provided some insight into how SR may lead to imbalances in the UPS system, and suggests that SR-exposure is associated with a pro-ubiquitination balance, whereas, in Sham rats, there is a pro-deubiquitination balance. However, this conclusion is based upon the very select protein signatures identified in this study, the importance of which to the “global” ubiquitination balance may be small. Specifically designed experiments utilizing ubiquitinated protein enrichment approaches would probably be necessary to definitively establish how SR exposure alters the functionality of the UPS system.

## 4. Materials and Methods

### 4.1. Irradiation Procedure

The rats used in this study are a subset of the 185 male Wistar retired breeder rats (HSD:WI; Harlan Sprague–Dawley, Inc., Indianapolis, IN, USA) that were used in our previous study [12]. The rats were delivered directly from the supplier to BNL, where they were group housed, maintained on a 12:12 light/dark cycle and given ad libitum access to autoclaved Purina Rodent Chow 5001 and municipal water by bottle. After at least one week of acclimatization, the rats were irradiated with 15 cGy 1 GeV/n ^48^Ti exposure at the NASA Space Radiation Laboratory at a dose rate of 10 cGy/min. After irradiation, the rats were implanted with ID-100us RFID transponders (Electronic ID Devices Ltd., Santa Barbara, CA, USA to facilitate identification of individual animals. One week after irradiation, the rats were transported to EVMS, where they were group housed, and given ad libitum access to Teklad 2014 rat chow and municipal water by bottle. The rats were maintained on a reversed 12:12 light/dark cycle, i.e., lights were switched off during working hours, resulting in the rats being in their active phase when tested for spatial memory performance.

### 4.2. Spatial Memory Testing

Spatial memory performance was established at 90 ± 14 days post irradiation. The rats were tested on the Barnes Maze in accordance with our previously published protocol [12]. A key element of the Barnes Maze is that rats progressively learn and recall the location of the escape box, as measured by the time to find its location i.e., Escape Latency. For each animal, an individualized metric of their relative increase (or decrease) in performance between days 1 and 3 of testing was calculated i.e., the Relative Escape Latency 3 (REL3), where the Day 3 ESL for a specific rat is expressed as a decimal fraction of its Day 1 ESL. The REL3 is less than unity if rats improve their spatial memory between days 1 and 3 of testing, but, if spatial memory is impaired, the REL3 remains at or above unity. 

### 4.3. Hippocampal Protein Extraction and Mass Spectrometric Profiling

The protocol followed for peptide and protein identification for the brain tissue lysate has been published in a previous paper [29]. Briefly, experiments were executed on the Q-Exactive (QE) mass spectrometer (Thermo Fisher Scientific, San Jose, CA, USA) using ‘bottom-up’ proteomics. A label-free precursor ion detection method (Proteome Discoverer, version 1.4, Thermo Scientific) was used because of the accurate mass measurements on proteins/peptides with specific retention times on precursors/fragments within 5 ppm mass accuracy. These factors combine to afford protein/peptide identifications with high confidence and high sequence coverage. The Sequest algorithm, a search engine employed by Proteome Discoverer (version 1.4, Thermo Scientific), was used to identify peptides from the resulting MS/MS spectra by searching against the combined Rat protein database (a total of 25,320 sequences) extracted from Swissprot (version 57) using taxonomy “Rattus”. Searching parameters for parent and fragment ion tolerances was set as 15 ppm and 80 mmu for the QE, and trypsin was set as the protease with a maximum of 2 missed cleavages. Only those proteins that have >2 peptides identified (or >50% of protein covered by a single peptide) were included in the comparative quantitative analysis steps, and result in a correct protein identification probability of *p* < 0.05.

Quantitation of a protein within a given technical replicate was achieved by calculating the area under the curve (AUC) for the respective de-isotoped peptide and charge reduced multiple tryptic peptides. A protein is classified as being “present” if it is identified in two of the three technical replicate samples for an individual rat hippocampus sample. In the event that a protein is not detectable in a particular cohort, an AUC value of 1 is assigned for that protein. The mean AUC value for each individual rat is calculated. A mean cohort AUC value (and the SEM) was then calculated for any protein that was “present” in the majority of the individual rats within that cohort. In those instances where the SEM exceeded the mean AUC, those proteins were removed from further analysis.

### 4.4. Post Analysis of the Proteins Identified by Proteome Discoverer

The online visual analysis tool Venny 2.1 [53] was used to establish protein signatures that were common to all cohorts, and those that were uniquely present in specific cohorts. Proteins that were up- or downregulated in the irradiated cohorts (i.e., all Ti exposed rats (TEM proteins), Ti-exposed rats that retained sham-like spatial memory performance (PSM proteins), and Ti-exposed rats that had impaired spatial memory performance (SMI proteins)) were then identified. The AUC values of the proteins in the irradiated cohorts were compared to the respective AUC values in the sham cohort and those proteins whose expression index (relative to the sham AUC value) was ≥1.5 (upregulated) or ≤0.66 (down-regulated) identified. The Wilcoxon–Mann–Whitney test was used to identify proteins whose expression differed from that seen in the sham-irradiated rats at the 5% significance level.

The functional significance of the protein signatures that characterized each of the phenotypic groups, (i.e., Shams, all Ti exposed rats, Ti-exposed rats that retained sham-like spatial memory performance and Ti-exposed rats that had impaired spatial memory performance) was established using the Compare gene enrichment function in FunRich version 3.1.3 [54], a freely available software tool. Prior to analyses, the Rodent (Taxon ID: 9989) database was downloaded from UniProt and configured as the background database within FunRich. The cohort protein signatures were characterized against known UniProt molecular functions and biological processes, with an inclusion criterion of ≥10% of proteins within the signature must be linked to that function, and that the function was identified in at least two of the four proteins (i.e., Sham, PSM, TEM, or SMI) signatures.

The online visual analysis tool Venny 2.1 [40] was used to identify the proteins within each signature that were present in the UniProt reference base for the UPS. A FunRich-generated report detailing the cellular component, molecular function, and biological process data of each protein was then generated. 

To provide some insight into the overall ubiquitination activity implicated by the various protein signatures, a simplistic ubiquitin homeostasis algorithm was developed. Signature proteins identified as being in the UniProt UPS database were assigned impact values based on how many and the types of ubiquitin-regulated functions they exhibited. Ubiquitination-promoting activity garnered positive scores, and ubiquitination-depressing (or deubiquitination) activity earned negative scores. For each instance wherein ubiquitination or deubiquitination was a primary function of that protein (that is, the protein is immediately responsible for ubiquitination or deubiquitination), they were given a +1 or −1 score, respectively. Non-primary functions were assigned +0.5 or −0.5; this includes any ubiquitination-adjacent function whose dysfunction does not immediately impact ubiquitination activity, but would likely lead to disruptions in the UPS (e.g., ubiquitin ligase activity, component of the 26S proteasome, positive or negative regulation of the catabolic process, etc.). Ambivalent proteins (i.e., proteins that were identified as overlapping with the UPS, but whose function or impact is not yet confirmed) were assigned a neutral 0. Once each value was assigned, scores were totaled by cohort in order to obtain a net impact value; positive scores indicate an abundance of ubiquitination activity, negative scores indicating less pro-ubiquitination activity. 

### 4.5. Statistical Analysis 

All statistical analysis was conducted using GraphPad Prism version 9.1.2 for Windows, GraphPad Software, San Diego, CA, USA, www.graphpad.com, assessed on 11 July 2021. 

## 5. Conclusions

In conclusion, this study has provided further insight into the complex changes that occur in the hippocampus of SR-exposed rats, and have identified specific protein signatures that characterize the hippocampus of rats that developed spatial-memory impairment as well as those that have managed to maintain sham-like performance levels. In addition, our study has demonstrated that there may be marked changes in the functionality of the UPS system that warrants further investigation, and specifically its role in regulating SR-induced neurocognitive impairment.

## Figures and Tables

**Table 1:** Sham Only signature proteins.

UniProt Accession Numbers §					
A1A5R8	P11951	P38438	Q4G017	Q6AXV4	Q8K4T4
B0BNE5	P12001 *	P53678	Q4KLZ4	Q6AY09 *	Q99MZ4
B2DD29	P13471 *	P55053	Q4VSI4 *	Q6AY30	Q99PF5
B2GV54	P15429	P56603	Q5FVQ8	Q6AY41	Q9ESB5
B2RZ37	P15431	P60892	Q5MJ12 *	Q6AYH5	Q9JHU0
B2RZ78	P15800	P62193 *	Q5U2Z5	Q6AYK6 *	Q9QYL8
D3ZEY4	P20069	P62824	Q62656	Q6MG06	Q9R085 *
D3ZGS3	P22062	P62959	Q62824	Q6MG49	Q9R0I8
D4ABY2	P23576	P68255 *	Q63009 *	Q6MG55	Q9R1N3
F1LSG8	P23965	P84586 *	Q63100	Q6MG60	Q9WU70
O35274	P26817	P86182 *	Q63633	Q6P7S1	Q9Z1C7
O54701	P29411	Q01066	Q63881	Q78P75	Q9Z269 *
P00564	P31422	Q02356	Q64232	Q7M6Z5	Q9Z2 × 5
P01026	P31424	Q1WIM2	Q64640	Q80W83 *	
P04256 *	P31977	Q4FZT0	Q66H20	Q811X6	

§ Fully mapped and annotated in UniProt. * Denotes matched to UPS data base.

**Table 2:** Titanium Exposure Marker (TEM) signature proteins.

UniProt Accession Numbers §			
F1LQ48 *	P13803	Q07303	Q4KLM4 *
O35763	P14604	Q02874	Q5M9I5
O70511	P18484	P62243 *	Q63803
P09330	P21396	P53676	Q68FS2 *
P13638	P26772	P48679 *	Q91XU8

§ Fully mapped and annotated in UniProt. * Denotes matched to UPS data base.

**Table 3:** Spatial Memory Impairment (SMI) signature proteins.

UniProt Accession Numbers §				
B0BN85 *	**P15178**	***P60901***	**Q4KM49**	*Q6P7Q4*
**B0BNF1**	*P15865*	*P61203*	**Q4V8I7**	*Q6QIX3*
D4AE41 *	**P18163**	*P61227*	**Q505J8**	*Q7TQ94*
F1LNJ2 *	P20171 *	**P62024**	**Q569C2**	*Q80W89*
F1M386 *	P21272 *	*P62250*	**Q5HZA6**	**Q8VHW5**
**O35179**	**P29101**	*P62914*	**Q5U216**	*Q9JHL4*
**O35314**	*P31647*	**P63045**	**Q5XIE8**	Q9JJ31 *
O35346 *	**P37285**	***P69682***	*Q5XIF3*	**Q9JKE3**
*P02650*	*P40307*	**P70619**	*Q5XIG8*	*Q9JMJ4*
*P04631*	*P47863*	*P84092*	*Q5XIT1*	*Q9WV63*
*P04785*	*P48768*	*Q08602*	Q62829 *	**Q9Z0W7**
P07153 *	*P51635*	**Q32ZG1**	**Q63635**	*Q9Z142*
*P09895*	*P55051*	***Q3B8Q0***	**Q64591**	**Q9Z1N1**
**P0C5X8**	**P56536**	*Q499P2*	**Q66X93**	
*P11232*	*P59215*	*Q4FZT2*	*Q68FP1*	

§ Fully mapped and annotated in UniProt. Bold font denotes only identified in this signature. Italic font denotes proteins that were unregulated (>1.5 fold) in this signature compared to shams. * Denotes matched to UPS data base.

**Table 4:** Preservation of Spatial Memory (PSM) signature proteins.

UniProt Accession Numbers §				
**B5DF41**	*P25093*	P62890 *	**Q3ZB98**	**Q8K4M9**
D3ZBM7 *	**P25809**	**P63029**	**Q497B0**	**Q91Z79**
**D4A6D8**	***P40329***	**P63088**	**Q4V882**	**Q99M64**
**O08557**	**P41123** *	**P69735**	*Q5M7U6*	**Q99PE7**
O35821 *	**P46101**	*P83868*	**Q5PQN0**	*Q9ER24*
**O54922**	**P46844**	*P84087*	**Q5U2T3**	*Q9JLA3*
**O55043**	**P47987**	**P85970**	**Q5XHY5**	*Q9QVC8*
P10686 *	**P61107**	**P97837**	**Q62717**	**Q9WTT2**
*P13264*	P62268 *	*Q05695*	**Q63228**	**Q9WVK7**
*P19468*	**P62853**	**Q07205**	**Q6RUV5**	*Q9Z0V6*
*P20650*	**P62859**	**Q07310**	**Q812D1**	*Q9Z270*

§ Fully mapped and annotated in UniProt. Bold font denotes only identified in this signature. Italic font denotes proteins that were unregulated (>1.5 fold) in this signature compared to shams. * Denotes matched to UPS data base.

**Table 5:** Relative abundance of signature proteins in the five operational GO processes (identified by FunRich analysis).

Go Process	Description	% Protein within Functional Signature Involved in the Process			
		Sham	TEM	SMI	PSM
0019901	Protein kinase binding.	3.75	16.67	9.09	13.89
0042802	Identical protein binding	21.25	11.11	30.3	8.3
0044877	Protein-containing complex binding	10.0	11.11	15.15	11.11
0046872	Metal ion binding	10.0	11.11	6.06	11.11
0005524	ATP binding	15.0	11.11	30.3	13.89

TEM: Titanium Exposure Marker; SMI: Spatial Memory Impairment; PSM: Preservation of Spatial Memory. Numbers represent the relative abundance of the unique signature proteins as a percentage of all proteins involved in the GO process.

**Table 6:** Abundance of Ubiquitin-related proteins in the protein signatures associated with various levels of spatial memory functionality.

Protein Signature Classification					
Sham	TEM	SMI	PSM	Impaired (TEM&SMI)	Functional (TEM&PSM)
15/88 (17%) §	5/20 (25%) §	10/37 (27%) §	7/41 (17%) §	15/61 (24.5%) §	12/57 (21.0%) §
P04256 P12001 P13471 P62193 P68255 P84586 P86182 Q4VSI4 Q5MJ12 Q63009 Q6AY09 Q6AYK6 Q80W83 Q9R085 Q9Z269	F1LQ48 P48679 P62243 Q4KLM4 Q68FS2	B0BN85 D4AE41 F1LNJ2 F1M386 O35346 P07153 P20171 P21272 Q62829 Q9JJ31	D3ZBM7 O35821 P10686 P41123 P62268 P62890 Q6RUV5	B0BN85 D4AE41 F1LNJ2 F1LQ48 F1M386 O35346 P07153 P20171 P21272 P48679 P62243 Q4KLM4 Q62829 Q68FS2 Q9JJ31	D3ZBM7 F1LQ48 O35821 P10686 P41123 P48679 P62243 P62268 P62890 Q4KLM4 Q68FS2 Q6RUV5

TEM: Titanium Exposure Marker; SMI: Spatial Memory Impairment; PSM: Preservation of Spatial Memory. § Numbers represent the number of URP proteins as a function of the total number of proteins found within the functional signature, and the calculated relative abundance of the Ubiquitin-related proteins (URP) proteins within each signature.

## Supplementary Materials

Supplementary Table S1 is available online at https://www.mdpi.com/article/10.3390/ijms22147713/s1.
